# Proceedings of the New York Central Homœopathic Society

**Published:** 1885-05

**Authors:** Julius Schmitt

**Affiliations:** Secretary pro tem.


### PROCEEDINGS OF THE NEW YORK CENTRAL HOMŒOPATHIC SOCIETY.

Dr. J. R. Young, the president, called the meeting to order at 10 A. M., in Dr. J. A. Biegler's office, Rochester, N. Y., on the 18th of December, 1884. After some routine business Dr. J. R. Young related a case of a young lady struck by lightning; any exposure to heat set her into nervous spasms, so that she had to go into a cool place. She was cured by Morphine 1x trituration once a day, taken for twelve consecutive days.

Dr. Julius Schmitt read a paper on the repetition of the dose in the treatment of chronic diseases, illustrating by the history of two cases the teachings of Hahnemann's in this respect.

Dr. R. R. Gregg reported a case of a horse, showing symptoms of spavin in winter-time for two years; then he got the pink-eye, which was cured by two doses of Arsenic (high); soon after this he broke out with a scaly eruption on the hip, disappearing in the course of two years. The lameness did not come on again and he was also cured of an easy tiring of the hind feet. Also, a case of a young lady of overtaxed brain and abdominal plethora, which called for several remedies, until a right-sided facial neuralgia appeared, which was cured by Spigelia (high). After this a scaly eruption broke out over the whole body and finally a paralysis of the left upper eyelid took place. All other symptoms relieved. The paralysis she had had once ten years ago.

He reported also a case of a horse with cracked hoof, cured by Graphites (high).

Dr. J. A. Biegler reported a case of an Indian missionary whose health was undermined by different diseases incidental to the hot climate and mismanaged by unscientific treatment. He grew very fleshy and had, three years ago, a hepatitis with right-sided pleuritis, with threatening of an abscess of liver. He was cured of this difficulty by Drosera$^{cm}$, given especially for the very constant symptom of pain in right hypochondrium, relieved by pressure, or lying on affected side with pillow bolstered under it. A European trip helped him greatly, and he remained apparently well, until lately, when the following symptoms appeared: *Dizziness, with a tendency to fall forward;* heaviness of legs, as if they were of lead; *increasing, enormous appetite, yet losing flesh all the time; intense thirst.* He received one dose of Iodium (high) and the urine was examined the following day—specific gravity, 1040; reaction, acid; sugar, six and sixty-six hundredths per cent.; urea, two per cent. This was on December 3d, 1884.

On December 17th, the urine was examined again, and, as in the first time, by Professor Lattimore. The result was the following: Specific gravity, 1020; reaction, acid; *sugar, none;* urea, three per cent. All the other symptoms are better also. All this time he had been allowed nothing but a strictly diabetic diet.

The Society adjourned at 1 A. M., accepting the amicable invitation of Dr. Biegler to a sumptuous lunch.

Society was called to order at 1.45 P. M.

Dr. Boyce reported a case of a woman who received for a chronic cough, with great chilliness as the most prominent symptom, one dose of Silicea$^{2c}$ (Dunham). She got better soon, but after three weeks another dose had to be given, which again helped her for a longer time, but a third dose was required to cure her. He enlarged then on the difficulty to tell how many doses of the curative remedy were required.

Dr. Gregg remarked that Dr. Adolph Lippe considers one powder dissolved in water and taken in short intervals as a single dose. He finds that repeated doses will generally produce new complications which are more serious than the one they have relieved. A cure may be always expected if, under the single dose, the disease changes from an important organ to a less important one. If it goes the other way, he is always afraid he has done harm.

Dr. Young recited his own case. He was kicked by a horse when seven years old. Since that time he suffered from a pain in chest; worse from any exertion, becoming at times almost unbearable. When a medical student, Professor Smith, of Chicago, cured him by one dose of Arnica (high). This was in 1872, and he had suffered since 1849.

Dr. Gregg reported a case of angina pectoris which resisted four doses of one-fourth grain of Morphine, given by an allopath, and was cured by a single dose of Belladonna$^{2000}$ in a very short time. The last dose of Morphia he had taken at 5 P. M., and the Belladonna was given at 8 P. M. Chief indication for the remedy was a *globular* pulse, which Dr. Gregg considers as one of the keynotes of Belladonna. He mentions also a case of scarlatina, which had been given up to die, saved by a single dose of Belladonna$^{2000}$, also given on account of the globular pulse being present. He describes it as if a shot were passing under the finger.

Dr. Young spoke of a case of convulsions recovering without any medicine.

Dr. Hawley reported his own case of zona on right side, pain going down to right leg, worse at nights, and necessitating continuous change of position. One dose of Rhus tox$^{75000}$ relieved markedly, but it got worse again, until he consulted Dr. Pierson, who gave him Rhus tox$^{75000}$ in water, which cured speedily.

Dr. Gregg mentioned the case of a lady who complained of pain in right leg and in calf of leg, *worse when coughing*, and from the slightest motion, better from warmth. Bryonia, Coloc., Bell., Phosph., Rhus, etc., were given all in the single dose without relief. Finally she was cured of it by a single dose of Nux vomica$^{2000}$. This same lady was cured in 1862 of a beginning phthysis pulmonum by a single dose of Lycopodium (high). She has had many different complaints, but was always relieved by the single dose of the curative remedy.

Dr. Boyce felt relieved that even Dr. Gregg does not always cure his patients by the first prescription.

Dr. Gregg thought there must be a possibility to find a key for the immediate knowledge of the right remedy.

Dr. Adams related a case of violent after-pains, with pain in left shin bone, cured by Carbo-vegetabilis.

Dr. Gregg: Platina has labor-pains all on the left side.

Dr. Hussey related a case of a young man with acute dyspepsia; food taken in the morning distresses him all day; very sour vomiting, especially after meals. Robinia relieved the acidity. He used to have an irritating condition of the scrotum every summer, until fourteen or fifteen years ago, after this all over the body. When eighteen years old he had an attack of boils in different parts of body. Sulphur (high) cured. The case shows that psora followed him through his whole life, assuming different forms of disease.

Dr. Schmitt reported cure of pain in the stump of an amputated finger, increased by breathing, with two doses of Phosphoric acid $^{cm}$.

Dr. Boyce mentioned a case of chronic headache, changing to dyspepsia; nightly pains; patient had been heavily dosed with Opium. After receiving Nux vomica, he gave the symptom of relief of pains by eating ice-cream. For this Phosphorus was given, then the symptoms changed to relief of pain by eating. Three pellets of Petroleum$^{2c}$ (Dunham) stopped the pain right off for three weeks, when another dose had to be given, which cured permanently.

Dr. Biegler cautioned against repeating the dose if there be a slight reverse of the symptoms.

Dr. Chaffee found more aggravations after high potencies than after the lower ones.

Dr. Biegler gave a case of pneumonia indicating Opium, which was given in the two hundredth potency for twenty-four hours without relief, when Opium$^{cm}$ helped.

Dr. Hawley reported a case of membranous croup cured by Hepar, first centesimal, after the thirtieth, two hundredth, and eighty-thousandth had failed. Also, a case of diphtheria with characteristic Hepar-cough, where Hepar, first centesimal, cured, after the various higher potencies had failed.

Dr. Biegler related the case of a gentleman rolling on the floor with violent gastralgia, crying for Morphine injections; indications being petulant bad temper. Chamomilla$^{cm}$, single dose, relieved in less than ten minutes.

Dr. Hawley moved the thanks of the Society be given to Dr. Biegler for his handsome entertainment, which, however, should not be considered as a precedent.

Dr. R. R. Gregg, of Buffalo, Dr. Allen B. Carr, and Dr. R. A. Adams, of Rochester, were proposed for membership, and accepted. They paid the initiation fee.

Dr. Boyce moved that the next meeting be held at Dr. Hawley's office, in Syracuse, inasmuch as some preparations have to be made for the meeting of the International Hahnemannian Association in June. Carried.

Dr. Boyce proposed to discuss at the next meeting the three precautions Hahnemann gives his followers in the treatment of chronic diseases, which was amended that Dr. Boyce should prepare a paper on this subject. Carried.

Dr. Hawley said that this Society has done him more good than any reading to understand Hahnemann.

Dr. Schmitt moved that the Secretary be instructed to send out the notices of meetings one month ahead. Adopted.

Adjourned at 4 P. M.

JULIUS SCHMITT, M. D.,
*Secretary pro tem.*

